# Publisher’s Note: A New Chapter for *Advances in Respiratory Medicine*—Continued Publication by MDPI

**DOI:** 10.3390/arm90040032

**Published:** 2022-07-13

**Authors:** Clàudia Aunós

**Affiliations:** MDPI, Av. Madrid 95, 1-3, 08028 Barcelona, Spain; claudia.aunos@mdpi.com

*Advances in Respiratory Medicine* is a journal with a rich history dating back to 1909, when the journal was previously named *Gruźlica (Tuberculosis)* and published in Polish. The publication was suspended in 1939 due to the Second World War and only resumed in 1947 [1].

In 1962, the journal underwent a transformation and became an affiliated journal of the Polish Phtisiopulmonology Society and the Institute of Tuberculosis and Lung Diseases [2] under the name *Gruźlica i Choroby Płuc*. In the early 1990s, the journal was renamed *Pneumologia i Alergologia Polska* [3] and became a bimonthly publication journal.

From 2015 onwards, all articles in the journal were published in English [4].

In 2016, the journal was renamed again to finally become the *Advances in Respiratory Medicine* that it is today [5]. Until June 2022, it was published by Via Medica [6], and as of Volume 90, Issue 4, *Advances in Respiratory Medicine* is published by MDPI [7]. It will continue to be an evolving and innovative journal, serving the scientific community by publishing high-quality, open access, and peer-reviewed articles on all aspects of diseases of the respiratory system.

We would like to offer a warm welcome to all Editorial Board Members, the Polish Respiratory Society members, and authors. We hope *Advances in Respiratory Medicine* will continue to serve as a platform for sharing advances in the field of respiratory medicine.
